# Prevalence and correlates of joint pain among Chinese breast cancer survivors receiving aromatase inhibitor treatment

**DOI:** 10.1007/s00520-022-07345-3

**Published:** 2022-09-06

**Authors:** Tao Wang, Yu-Yan Huang, Xian-Liang Liu, Alex Molassiotis, Li-Qun Yao, Si-Lin Zheng, Jing-Yu (Benjamin) Tan, Hou-Qiang Huang

**Affiliations:** 1 College of Nursing and Midwifery, Charles Darwin University, 410 Ann Street, Brisbane, QLD 4000 Australia; 2 School of Nursing, Southwest Medical University, 319 Zhongshan Rd 3rd Section, Luzhou, 646000 Sichuan China; 3 School of Nursing, The Hong Kong Polytechnic University, Kowloon, Hong Kong SAR; 4 The Affiliated Hospital of Southwest Medical University, 25 Taiping Street, Luzhou, 646000 Sichuan China

**Keywords:** Breast cancer survivors, Pain, Musculoskeletal symptoms, Aromatase inhibitors, Cross-sectional study

## Abstract

**Background:**

Aromatase inhibitor (AI)-induced joint pain is a common toxicity of AI treatment. Although many studies have been conducted to examine the occurrence and severity of AI-induced joint pain in breast cancer survivors, none of the studies focused on the Chinese population with breast cancer. Given that the differences in cultural background and the genetic structure between Asians and Caucasians may contribute to different phenotypes of joint pain, this cross-sectional study was therefore conducted to examine the prevalence of AI-induced joint pain among Chinese breast cancer survivors receiving AI treatment and the correlates of pain.

**Methods:**

This cross-sectional study was conducted in a tertiary hospital in China. Breast cancer survivors undergoing AI treatment were recruited to complete the following questionnaires: a self-designed baseline data form, the Nordic Musculoskeletal Questionnaire (NMQ), the Brief Pain Inventory (BPI), the 36-Item Short Form Health Survey (SF-36), and the Functional Assessment of Cancer Therapy-Breast (FACT-B). Based on the assessment results of NMQ (if the participant indicated pain in specific body parts), participants were then invited to complete other questionnaires to specifically assess the joint symptoms, including the Oxford Knee Score (OKS), the Oxford Hip Score (OHS), the Michigan Hand Outcomes Questionnaire (MHQ), and the Manchester Foot Pain Disability Questionnaire (MFPDQ). Descriptive analysis was used to analyse participants’ baseline data and the prevalence of pain. Stepwise multiple regression was used to identify the correlates of pain.

**Results:**

Four hundred and ten participants were analysed. According to the NMQ, 71.7% of the participants experienced joint symptoms in at least one joint, and the most frequently mentioned joint was knee (39.0%). The diagram in BPI indicated that 28.0% of the participants had the worst pain around knees. In patients with knee pain, the mean OKS score was 40.46 ± 6.19. The sub-scores of BPI for pain intensity and pain interference were 1.30 ± 1.63 and 1.24 ± 1.79, respectively. Patients’ poorer physical well-being/functioning, previous use of AI treatment, presence of osteoarthritis, and receiving of physiotherapy were identified as four common correlates of greater severity of pain and pain interference (*p* < 0.05).

**Conclusions:**

Chinese breast cancer survivors can experience joint pain at various locations, particularly knees. In addition to increasing the use of interventions for pain alleviation, a comprehensive assessment of survivors’ conditions such as physical functioning, history of AI treatment, and presence of osteoarthritis should be emphasized to identify survivors who need more attention and tailored interventions.

## Introduction

The GLOBOCAN 2020 estimated that female breast cancer is the most prevalent cancer worldwide, with an estimated 2.3 million new cases in 2020 [1]. A similar trend was found in China, where breast cancer is the most common cancer in women, with an incidence of 42.67 per 100,000 [2]. Oestrogen exposure is an important cause of breast cancer, and approximately 60–70% of the patients with breast cancer are oestrogen receptor (ER) positive [3]. Although deaths related to breast cancer have been decreasing due to the advances in screening and treatments, breast cancer survivors still experience a wide range of side effects caused by pharmacological treatment [4]. Aromatase inhibitors (AIs) are an effective and commonly used drug in treating ER-positive breast cancer [3, 4]. However, AIs can cause many side effects such as joint pain, cardiovascular events, and a decrease in cognition functioning [3]. Of which, joint pain has emerged as a major side effect of AI treatment, and it can impact not only the survivors’ lifestyles but also lead to AI discontinuation [4].

Although a full understanding of the multifactorial biological mechanism underlying AI-induced joint pain is still lacking [5], a consensus regarding the key features of AI-induced joint pain has been achieved: it happens in breast cancer patients who are currently receiving AI treatment, the joint pain happened or worsened since the AI treatment, and the pain can be improved within 2 weeks after interruption of AIs but returns upon resuming AIs [6]. It has been demonstrated that approximately 50% of the breast cancer survivors receiving AI treatment experience joint pain within the first half-year of treatment, and up to 20% of those survivors tend to discontinue due to the joint pain [7–9]. Early discontinuation of the AI treatment can further increase the likelihood of recurrence and thus decrease the survival time of survivors [10]. A cohort study indicated that non-adherence to endocrine therapy including AI treatment can increase the risk of recurrence by up to 71% and double the risk of all-cause death [11].

Recent pharmacogenetics and pharmacogenomics suggested that the genetic and genomic variation at the population level is associated with the drug metabolism of and drug response to AIs, which may contribute to variability in the phenotype (e.g. occurrence, severity) of joint pain [12]. A full understanding of the phenotype and pre-treatment associates of AI-induced joint pain in breast cancer survivors can contribute to enhanced symptom monitoring and management programmes, as well as more tailored and specific healthcare services or interventions [13]. Many studies [14–18] have been conducted to examine the occurrence and severity of AI-induced joint pain, as well as the correlates of AI-induced joint pain in breast cancer survivors. One study [14] on White breast cancer patients reported that 52% of them experienced AI-induced joint pain, and their joint pain was associated with not only treatment-related factors (e.g. duration of AI treatment and history of chemotherapy) but also genetic factors (the nucleotide polymorphisms (SNP) rs11648233 within the HSD17B2 gene). None of the studies focused on the Chinese population with breast cancer. Besides, a systematic review [18] reported that some methodological limitations were found in the previous cross-sectional studies, for example, unclear descriptions of the study settings, participants, and statistical methods, which can affect the reliability and generalizability of the study findings in other contexts. Pain is a very complex experience that is associated with different factors, including not only the disease and treatment itself but also individuals’ own characteristics such as their sociocultural background [19]. The genetic structure between Asians and Caucasians is different [20], which may contribute to different phenotypes of joint pain such as the prevalence and the severity of pain. It is therefore inappropriate to develop healthcare services or interventions for Chinese patients that are directly based on the research evidence generated from other ethnicities. Hence, this cross-sectional study was conducted to examine the prevalence and relevant correlates of AI-induced joint pain among Chinese breast cancer survivors undergoing AI treatment.

## Methods

### Study design

The study was a cross-sectional study conducted in a tertiary hospital in China from April 2019 to January 2021. A pre-print version of this manuscript is available at https://assets.researchsquare.com/files/rs-1390711/v1/e6c693d2-e817-4efa-b7fb-4040dd0555bb.pdf?c=1646771430.

### Sample and sample size calculation

Inclusion criteria include (1) adult women with a confirmed breast cancer diagnosis at stage I, II, or IIIa; (2) had completed different types of cancer chemotherapy and currently undergoing AI treatment for over three months; (3) have a primary school education or above and are able to communicate in Chinese; and (4) agree to participate in the survey with written informed consent.

Sample size was calculated using the formula of *n* = (*Z*^2^ *P* (1 − *P*))/*d*^2^ (*Z* = statistic for a level of confidence, *P* = expected prevalence, and *d* = precision) [19]. The expected prevalence of joint pain in breast cancer survivors was estimated based on a previous study, where the prevalence of joint pain was 62% [21]. Therefore, a “*P*” value of 0.62 was utilized. Considering at least 10% of missing value or non-response, the final sample size was estimated as at least 402.

### Instruments

#### Baseline data assessment form

A self-developed form was used to collect participants’ demographic and clinical characteristics such as age, body mass index (BMI), years since last menstrual period, and cancer stage.

#### Nordic Musculoskeletal Questionnaire (NMQ) Modified Version

The NMQ is specifically designed for assessing musculoskeletal symptoms with well-documented psychometric properties [22]. The first part of the NMQ modified version presents a figure of human body and highlights nine body areas for pain assessment, while the second part was designed for evaluating the presence of pain in the areas and the impact of pain on daily activities [22]. The validated simplified Chinese version of the NMQ was used in this study [23] and the Cronbach’s alpha for the current sample was 0.81.

#### Brief Pain Inventory (BPI)

The validated simplified Chinese version of BPI was used in this study, which is a commonly used instrument for pain assessment in cancer population [24]. It has 15 items and mainly assesses the locations of pain, the intensity of pain during the past 24 h, and the impact of pain on daily functions [24]. A higher score indicates more severe pain/interference. In this sample, Cronbach’s alpha of pain intensity and pain interference were 0.89 and 0.95, respectively.

#### Assessment for joint symptoms identified in particular parts of the body

If participants indicated pain in specific body parts in NMQ, they were then invited to complete the following scales to specifically assess the joint symptoms.

##### Oxford Knee Score (OKS)

The OKS was specifically developed to assess the pain and function in knees [25]. It has 12 items and has been well validated in Chinese [26]. A higher score indicates a better outcome. In this sample, Cronbach’s alpha was 0.90.

##### Oxford Hip Score (OHS)

The OHS is a 19-item questionnaire for assessing hip pain [27]. A higher score indicates a better outcome. The simplified Chinese version of the OHS was used in this study, with the Cronbach’s alpha of 0.83 in this sample.

##### Michigan Hand Outcomes Questionnaire (MHQ)

The Chinese version of the MHQ has 37 items and six dimensions: overall hand function, activities of daily living (ADL), work, pain, aesthetics, and satisfaction assessing [28]. Except for the pain scale, higher scores indicate better performance for all other five scales. The Cronbach’s alpha for this sample was 0.83.

##### The Manchester Foot Pain Disability Questionnaire (MFPDQ)

The simplified Chinese version of the MFPDQ (19 items) was used in this survey, with the Cronbach’s alpha of 0.92 in this sample.

##### Functional Assessment of Cancer Therapy-Breast (FACT-B)

The FACT-B is a specific quality of life (QoL) scale for breast cancer patients. The FACT-General part includes 27 items with four domains including physical, social/family, emotional, and functional well-being [29]. The part of breast cancer module (BCS) has 10 items [29]. A higher score indicates better well-being. Cronbach’s alpha for the current sample was 0.90.

##### The 36-Item Short Form Health Survey (SF-36)

The SF-36 has 36 items with eight domains: “physical functioning”, “bodily pain”, “role limitation due to physical health problems”, “role limitations due to emotional problems”, “emotional well-being”, “social functioning”, “energy/fatigue”, and “general health perception” ([30], p. 1). A higher score indicates a more favourable health status. Cronbach’s alpha for the current sample was 0.91.

## Statistical analysis

SPSS 26.0 was used for data analysis. Descriptive analysis was used to present sample characteristics including demographic and clinical characteristics as well as the characteristics of pain and QoL. Inferential statistics were used to identify the correlates of pain severity and pain interference. For categorical variables such as education level, univariate analysis was conducted to identify the potential significant variables. For continuous variables such as age, correlation analysis was employed to identify the potential significant variables. Prior to the univariate analysis and correlation analysis, normality of the data was examined using Shapiro–Wilk for categorical variables and skewness and kurtosis for continuous variables [31]. According to the results of the normality test, non-parametric tests (three or more groups, Kruskal–Wallis test; two groups, Mann–Whitney test) were utilized for univariate analysis, and Pearson correlation was used for correlation analysis. Variables that showed statistical significance (p < 0.05) in the univariate analysis and correlation analysis were included in the stepwise multiple regression analysis to quantify the unique contribution of each potential variable to the survivors’ joint pain.

## Results

### Characteristics of the participants

Data from four hundred and ten participants were analysed in this study. The mean age of the participants was 53.87 years (SD = 8.28). All the participants were receiving AI treatment and the mean duration of the AI treatment was 27.31 months (SD = 21.20). All the participants have completed the chemotherapy, with a mean duration of 4.54 months (SD = 1.37). Most participants (83.8%) were postmenstrual women, with the mean years since the last menstrual period being 7.57 years (SD = 6.08). The mean BMI of the participants was 23.93 (SD = 3.22). More than 60% of the participants were at stage II breast cancer. More demographic and clinical characteristics are presented in Table 1.

**Table 1:** Demographic and clinical characteristics of the participants

Variables		Number (percentage %)
Education background (*n* = 410)	Primary school	161 (39.3)
	Secondary school	159 (38.8)
	High school	47 (11.5)
	Diploma	20 (4.9)
	Bachelor or above	23 (5.6)
Marital status (*n* = 405)	Unmarried	34(8.4)
	Married/single	371 (91.6)
Occupation (*n* = 407)	Professionals	13 (3.2)
	Labours	53 (13.0)
	Homemaker	126 (31.0)
	Clerical/administrative staff	18 (4.4)
	Others	86 (21.1)
	Unemployed	19 (4.7)
	Retired	92 (22.6)
Family income (yuan/month) (*n* = 382)	< 3000	158 (41.4)
	3000–6000	132 (34.6)
	> 6000–10,000	65 (17.0)
	> 10,000	27 (7.1)
Cancer stage (*n* = 410)	I	85 (20.7)
	IIa	158 (38.5)
	IIb	108 (26.3)
	IIIa	59 (14.4)
Osteoarthritis (*n* = 396)	Yes	30 (7.6)
	No	366 (92.4)
Previous chemotherapy regimens (*n* = 408)	Taxane-based	336(82.4)
	Not taxane-based	62(15.2)
	Unspecified	10(2.5)
Using of non-steroidal anti-inflammatory drugs (*n* = 391)	Yes	4 (1.0)
	No	385 (98.5)
	Unclear/unknown	2 (0.5)
Using of acetaminophen (*n* = 390)	No	389 (99.7)
	Unclear/unknown	1 (0.3)
Using of opioids (*n* = 390)	Yes	1 (0.3)
	No	388 (99.5)
	Unclear/unknown	1 (0.3)
Currently receiving physiotherapy (*n* = 400)	Yes	30 (7.5)
	No	369 (92.3)
	Unclear/unknown	1 (0.3)
Currently receiving massage therapy (*n* = 395)	Yes	20 (5.1)
	No	374 (94.7)
	Unclear/unknown	1 (0.3)
Lymphedema (*n* = 406)	Yes	24 (5.9)
	No	382 (94.1)
Insomnia (*n* = 408)	Yes	255 (62.5)
	No	152 (37.3)
	Unclear/unknown	1 (0.2)
History of AI treatment (*n* = 394)	Yes	64 (16.2)
	No	330 (83.8)
Duration of daily physical exercise (per week) (*n* = 405)	0–2 h	99 (24.4)
	3–4 h	33 (8.1)
	5–6 h	23 (5.7)
	> 6 h	250 (61.7)

### Joint pain in breast cancer survivors receiving AI treatment

#### Overall joint symptoms measured by NMQ

Two hundred and ninety-four participants (*n* = 294/410, 71.7%) experienced joint symptoms for at least one joint. The most frequently mentioned joint was knee (*n* = 160/410, 39.0%), which was followed by wrists/hand (*n* = 97/410, 23.7%), shoulders (*n* = 97/410, 23.7%), and lower back (*n* = 50/410, 12.2%). More details about other joints are presented in Table 2.

**Table 2:** Overall joint symptoms measured by NMQ (*n* = 410)

Pain locations (number, %)		Q1: have you at any time during the last 12 months had trouble (such as ache, pain, discomfort, numbness) in:	Q2: during the last 12 months have you been prevented from carrying out normal activities (e.g. job, housework, hobbies) because of this trouble in:	Q3: during the last 12 months have you seen a physician for this condition:	Q4: during the last 7 days have you had trouble in:
		With the answer of “yes”, number (percentage %)			
Ankles/feet	37 (9.0)	*n* = 21 (5.1)	*n* = 6 (1.5)	7 (1.7)	17 (4.1)
Elbows	24 (5.9)	*n* = 16 (3.9)	*n* = 7 (1.7)	4 (1.0)	15 (3.7)
Hips/thighs	19 (4.6)	*n* = 11 (2.7)	*n* = 6 (1.5)	6 (1.5)	9 (2.2)
Upper back	20 (4.9)	*n* = 9 (2.2)	*n* = 1 (0.2)	2 (0.5)	9 (2.2)
Knees	160 (39.0)	*n* = 125 (30.5)	*n* = 37 (9.0)	30 (7.3)	103 (25.1)
Lower back	50 (12.2)	*n* = 30 (7.3)	*n* = 12 (2.9)	10 (2.4)	27 (6.6)
Neck	41 (10.0)	*n* = 28 (6.8)	*n* = 7 (1.7)	10 (2.4)	23 (5.6)
Shoulders	97 (23.7)	*n* = 65 (15.9)	*n* = 20 (4.9)	20 (4.9)	55 (13.4)
Wrists/hands	97 (23.7)	*n* = 71 (17.3)	*n* = 20 (4.9)	9 (2.2)	62 (15.1)

*NMQ*, Nordic Musculoskeletal Questionnaire (NMQ) Modified Version

#### Joint pain in particular locations: knees, hands, feet, and hips

One hundred and fifty-five of the participants who experienced knee symptoms (measured by NMQ) further completed the OKS scale, with the mean OKS total score being 40.46 (SD = 6.19). For the mean score of each OKS item, the lowest score (mean = 2.20) was identified for item 1 (usual pain in the knee) on a 0 to 4 rating scale, with a higher score indicating a better outcome. For the other 11 items regarding trouble in performing daily activities due to knee pain (i.e. walking), the mean score ranged from 3.23 to 3.84. Details about the score of each OKS item are separately reported in another paper [32]. One hundred participants who experienced hand symptoms (measured by NMQ) further completed the MHQ, with the mean score of each subscale being 59.58 (SD = 16.14, overall hand function), 94.07 (SD = 11.04, overall ADL), 70.90 (SD = 25.03, work performance), 24.54 (SD = 21.73, pain), 80.18 (SD = 17.49, aesthetics), and 45.67 (SD = 18.36, satisfaction with hand function). Only 30 and 17 participants completed the MFPDQ and OHS to further assess the pain at the feet and hip, with a mean total score of 13.03 (SD = 9.33) and 39.41 (SD = 5.12), respectively.

#### Overall joint pain severity and pain interference

The diagram of the BPI indicated that more than half of the participants (*n* = 229/410, 55.9%) experienced pain in at least one location. One hundred and fifteen participants (*n* = 115/410, 28.0%) reported that the pain was mainly located around knee, which was followed by the locations around the shoulder (*n* = 73/410, 17.8%) and wrists and hand (*n* = 72/410, 17.6%) and low back (*n* = 41/410, 10.0%). As shown in Table 3, the mean scores for the subscale of pain intensity and pain interference were 1.30 (SD = 1.63) and 1.24 (SD = 1.79), respectively.

**Table 3:** Pain severity and pain interference measured by BPI

BPI pain severity during the past 24 h	*N*	Mean ± SD
(1) Worst pain in the past 24 h	410	2.44 ± 2.73
(2) Least pain in the past 24 h	410	0.48 ± 1.07
(3) Average pain in the past 24 h	410	1.45 ± 1.83
(4) How much pain you have right now	409	0.84 ± 1.54
Subscale score of pain severity	409	1.30 ± 1.63
BPI pain interference on daily functions		
General activity	408	1.60 ± 2.18
Mood	408	1.52 ± 2.22
Walking ability	406	1.03 ± 2.03
Normal work (includes both work outside the home and housework)	408	1.33 ± 2.18
Relation with other people	408	0.73 ± 1.71
Sleep	408	1.17 ± 2.06
Enjoyment of life	407	1.29 ± 1.95
Subscale score of pain interference	405	1.24 ± 1.79

*BPI*, Brief Pain Inventory

#### Quality of life

The mean total FACT-B score was 115.12 (SD = 16.41). The highest sub-score of SF-36 was physical functioning (86.89 ± 14.61). More details about the score of quality of life are shown in Table 4.

**Table 4:** Quality of life measured by FACT-B and SF-36

Quality of life	*N*	Mean ± SD
FACT-B		
Physical well-being	410	24.09 ± 3.92
Social/family well-being	410	22.22 ± 5.41
Emotional well-being	410	19.78 ± 3.96
Functional well-being	410	19.18 ± 5.36
Breast cancer subscale	410	29.85 ± 4.14
Total score of FACT-B	410	115.12 ± 16.41
SF-36		
Physical functioning	405	86.89 ± 14.61
Role physical	407	62.84 ± 44.97
Bodily pain	410	68.71 ± 17.85
General health	407	57.46 ± 17.57
Vitality	409	75.77 ± 13.52
Social functioning	410	85.31 ± 15.67
Role emotional	409	70.25 ± 42.95
Mental health	408	77.36 ± 13.40

*FACT-B*, Functional Assessment of Cancer Therapy-Breast; *SF-36*, the 36-Item Short Form Health Survey

### Factors linked to the joint pain severity and pain interference

#### Univariate analysis

For pain intensity, statistically significant variables of more severity of pain that were identified in univariate analysis were family income, presence of osteoarthritis, duration of daily physical exercise, receiving physiotherapy, and receiving massage (*p* < 0.05). For pain interference, the following variables were identified as significant variables of greater pain interference, including the presence of osteoarthritis, previous use of AI treatment, duration of daily physical exercise, receiving physiotherapy, and receiving massage (*p* < 0.05).

#### Correlation analysis

The correlation analysis indicated that breast cancer survivors’ pain severity was negatively correlated with all the sub-scores and total score of FACT-B (*r* =  − 0.222 ~  − 0.683) and SF-36 (*r* =  − 0.316 ~  − 0.715). Pain interference was positively correlated with age (*r* = 0.119) and years since the last menstrual period (*r* = 0.116) but negatively correlated with all the sub-scores of FACT-B (*r* =  − 0.156 ~  − 0.690) and SF-36 (*r* =  − 0.337 ~  − 0.657) (Table 5).

**Table 5:** Correlations between participants’ age, BMI, menopause time, AI treatment time, chemotherapy duration, FACT-B, SF-36, and BPI

Variables	BPI	
	Pain severity	Pain interference
Age	0.088	0.119*
BMI	0.018	0.043
Years since last menstrual period	0.072	0.116*
Duration of AI treatment time	0.009	0.009
Duration of completed chemotherapy	0.008	− 0.001
Physical well-being	− 0.683**	− 0.690**
Social/family well-being	− 0.222**	− 0.156**
Emotional well-being	− 0.375**	− 0.365**
Functional well-being	− 0.323**	− 0.312**
Breast cancer subscale	− 0.424**	− 0.396**
FACT-B total	− 0.539**	− 0.506**
Physical functioning	− 0.470**	− 0.489**
Role physical	− 0.471**	− 0.497**
Bodily pain	− 0.715**	− 0.657**
General health	− 0.403**	− 0.382**
Vitality	− 0.370**	− 0.385**
Social functioning	− 0.452**	− 0.462**
Role emotional	− 0.330**	− 0.355**
Mental health	− 0.316**	− 0.337**

*BMI*, body mass index; *FACT-B*, Functional Assessment of Cancer Therapy-Breast. *SF-36*, the 36-Item Short Form Health Survey. *BPI*, Brief Pain Inventory. **Correlation is significant at the 0.01 level (2-tailed). *Correlation is significant at the 0.05 level (2-tailed)

#### Regression analysis: correlates of joint pain

Regression analysis in Table 6 showed that breast cancer survivors’ poorer bodily pain, poorer physical well-being, receiving physiotherapy, and presence of osteoarthritis were the four important variables in predicting a greater severity of pain (*p* < 0.05). All those four factors together explained 61.5% of the survivors’ pain severity. For pain interference, the poorer physical well-being/function, poorer bodily pain, presence of osteoarthritis, receiving physiotherapy, previous use of AI treatment, and poorer role limitation due to physical health problems were identified as the important variables in predicting a greater pain interference (*p* < 0.05). All those identified factors together explained 58.1% of the survivors’ pain interference.

**Table 6:** Stepwise multiple linear regression for variables predicting pain severity and interference

BPI	Variables	Std. error	β	*t*	*p*	*R*^2^	Adj. *R*^2^
Pain severity	Bodily pain	0.004	− 0.435	− 9.786	.000	0.615	0.610
	Physical well-being	0.018	− 0.365	− 8.325	.000		
	Physiotherapy	0.260	− 0.139	− 4.139	.000		
	Osteoarthritis	0.201	− 0.086	− 2.598	.010		
Pain interference	Physical well-being	0.025	− 0.317	− 5.941	0.000	0.581	0.570
	Bodily pain	0.006	− 0.242	− 4.238	0.000		
	Osteoarthritis	0.256	− 0.172	− 4.380	0.000		
	Physical functioning	0.006	− 0.146	− 2.996	0.003		
	Physiotherapy	0.326	− 0.123	− 3.025	0.003		
	History of AI treatment	0.184	− 0.102	− 2.592	0.010		
	Role physical	0.002	− 0.098	− 2.077	0.039		

## Discussion

Considering the different genetic structures in different ethnicities, understanding the AI-induced joint pain in Chinese breast cancer survivors and its correlates can help clinicians to prioritize tailored health services or interventions for pain relief [11]. According to a systematic review on the prevalence of AI-induced joint pain [18], a very small number of studies (*n* = 4/21) reported the anatomical location of AI-induced pain. In this sample, the specific locations of the participants’ joint pain were identified, and knee was reported as the most prevalent pain location (39.0%). This finding was supported by a previous study [33], in which knee was reported as the most affected joint of AI treatments with the percentage of 33.1%. For the prevalence of AI-induced joint symptoms in different locations in this sample, the percentages ranged from 4.6 to 39.0% (measured by the NMQ), which were significantly different from other studies (ranging from 20.0 to73.7%) [18]. Such a high heterogeneity might be caused by the variety of instruments used for pain measurement in different studies. Some studies used BPI [34], while some others used instruments like the visual analogue scale [35] and functional assessment of cancer therapy-endocrine subscale (FACT-ES) [36]. These scales have different assessment time frames; for instance, the BPI is an instrument used to assess pain in the past 24 h; however, the FACT-ES assess pain in the past 7 days. The mean score of pain severity in this sample was rated as 1.30, which was lower than a previous study that used the same instrument (BPI) to measure pain of metastatic breast cancer patients (mean = 2.23) [37]. One possible explanation might be that the participants in this study were breast cancer survivors at an early stage and had completed the active anti-cancer treatments including chemotherapy, while pain is more frequent and severe for cancer patients who are at an advanced stage [38]. Besides, the ethnic difference and genetic variations between the study samples in the two studies (the previous study [37] recruited participants in the USA) might also lead to different study findings as genetic variation involved in metabolism of AIs may contribute to the variations in the occurrence and severity of the side effects including joint pain [12].

Survivors’ poorer physical well-being/functioning, previous use of AI treatment, presence of osteoarthritis, and receiving physiotherapy were identified as the common and significant correlates for high level of pain severity and pain interference. AI-induced joint pain can negatively impact survivors’ walking and other physical functions [39]. Previous research evidence indicated that breast cancer survivors who had a poorer physical function of lower extremities, shoulders, and hands since the AI treatment were more likely to report reductions in physical activities [40]. An increasing body of evidence has demonstrated that physical exercise such as walking and strength training can in turn increase the physical and functional well-being of survivors with joint symptoms [41] as well as effectively relieve the joint pain of breast cancer survivors undergoing AI treatment [42]. AI-induced joint pain, poorer physical, and functional well-being, and participation in physical exercise are therefore interrelated to each other, which can help explain why the breast cancer survivors with poorer physical well-being/functioning reported greater severity of pain. However, due to the nature of the cross-sectional study, causality among AI-induced joint symptoms, physical well-being, and participation in physical exercise cannot be determined in this study, which can be further explored in future research via a longitudinal study.

Participants’ AI treatment history was identified as another factor associated with the greater interference of joint pain. In this study, 16.2% of the participants had previously received AI treatment prior to the current course of AI treatment. This study finding can be partly supported by previous study findings that breast cancer survivors who previously received hormone replacement therapy (HRT) were more likely to develop musculoskeletal symptoms than those without previous HRT use [43]. The presence of osteoarthritis was identified as another significant correlate of AI-induced joint pain in this study. The possible explanation might be that both the AI-induced joint pain and osteoarthritis are believed related to oestrogen deprivation which can increase chronic inflammatory conditions and lead to pain particularly in joints [44, 45]. Interestingly, participants who were receiving physiotherapy reported greater severity of pain. However, the study finding should be interpreted with caution, and physiotherapy cannot be concluded as a risk factor for joint pain. According to previous studies, it is common that people use problem-focused coping strategies to deal with problems and distress actively by seeking advice and help from others [46]. Thus, one possible explanation for this finding might be that patients who had more severity of pain were more active regarding help-seeking, and physiotherapy was one of the treatments that they preferred to approach. Whether any biological mechanism/pathways exist between physiotherapy and AI-induced joint pain cannot be concluded through this study but is worthy of further exploration in future studies by adopting some biomarkers [5] such as receptor activator of nuclear factor-kB ligand (RANKL) as the potential indicators.

## Implications and limitations

Study findings indicated that, in clinical practice, in addition to increasing the use of interventions for pain alleviation, there is a need to comprehensively assess the clinical characteristic of breast cancer survivors such as previous use of AI treatment and presence of osteoarthritis to identify the target group for more specific attention. Considering the high incidence of pain in the knees and the negative influence of poor physical well-being/functioning on pain, some specific components can be included in the intervention programme to make it more tailored and effective, for example, promoting survivors’ understanding of the importance of physical exercise via health education and providing more upper extremity exercises or low intensity lower extremity exercises. In future research, more studies should be conducted to explore the correlates of AI-induced joint pain in Chinese breast cancer survivors at a genetic level.

This study has some limitations. The study was conducted at one study site using convenience sampling, which might limit its representativeness and the generalizability of the study findings. There might be a risk of over-analysing the results as multiple testing was conducted in this study although only subgroups with relatively adequate sample size were analysed. Although the knee, shoulder, and hand were identified as the top three joint pain locations, further sub-group analyses based on these three locations (e.g. levels of pain in three groups) were not conducted as some patients reported pain at more than two joints which made it difficult to separate the participants into three different groups. Physiotherapy was identified as one of the correlates of joint pain; however, further sub-group analyses based on the types and dose of physiotherapy (e.g. frequency and duration) could not be conducted due to the absence of relevant information. In addition, although most of the participants were postmenopausal patients, there were still a small number of premenopausal or perimenopausal patients who might receive AI treatment in combination with ovarian function suppression; however, relevant treatment information regarding ovarian function suppression in those premenopausal or perimenopausal patients was not collected as a confounding factor to determine to which extent the joint pain was related to the ovarian function suppression.

## Conclusion

Chinese breast cancer survivors experienced various types of joint pain, particularly knee pain. The importance of identifying correlates of joint pain such as survivors’ poorer physical functioning, their previous use of AI treatment, and the presence of osteoarthritis should be recognized and assessed comprehensively in clinical practice to identify the target group for more attention as well as to inform the development of individualized health services or interventions.

## Data Availability

Datasets used and analysed in this study are available from the corresponding author on a reasonable request basis.
